# Risk factor analysis of intravesical recurrence after retroperitoneoscopic nephroureterectomy for upper tract urothelial carcinoma

**DOI:** 10.1186/s12894-021-00932-2

**Published:** 2021-12-02

**Authors:** Masato Yanagi, Tsutomu Hamasaki, Jun Akatsuka, Yuki Endo, Hayato Takeda, Yukihiro Kondo

**Affiliations:** 1 Department of Urology, Nippon Medical School Hospital, 1-1-5, Sendagi, Bunkyo-ku, Tokyo 113-8603 Japan; 2 Department of Urology, Nippon Medical School Musashikosugi Hospital, 1-396, Kosugityo, Nakahara-ku, Kawasaki city, Kanagawa 211-8533 Japan

**Keywords:** Upper urinary tract, Urothelial carcinoma, Retroperitoneoscopic nephroureterectomy, Pneumoretroperitoneum time, Intravesical recurrence, Urine cytology

## Abstract

**Background:**

One of the major concerns of patients with upper tract urothelial carcinoma (UTUC) treated with nephroureterectomy is intravesical recurrence (IVR). The purpose of the present study was to investigate the predictive risk factors for IVR after retroperitoneoscopic nephroureterectomy (RNU) for UTUC.

**Methods:**

Clinicopathological and surgical information were collected from the medical records of 73 patients treated with RNU for non-metastatic UTUC, without a history of or concomitant bladder cancer. The association between IVR after RNU and clinicopathological and surgery-related factors, including preoperative urine cytology and pneumoretroperitoneum time, was analyzed using the Fisher exact test.

**Results:**

During the median follow-up time of 39.1 months, 18 (24.7%) patients had subsequent IVR after RNU. The 1- and 3-year IVR-free survival rates were 85.9% and 76.5%, respectively. The Fisher exact test revealed that prolonged pneumoretroperitoneum time of ≥ 210 min was a risk factor for IVR in 1 year after RNU (*p* = 0.0358) and positive urine cytology was a risk factor for IVR in 3 years after RNU (*p* = 0.0352).

**Conclusions:**

In UTUC, the occurrences of IVR in 1 and 3 years after RNU are highly probable when the pneumoretroperitoneum time is prolonged (≥ 210 min) and in patients with positive urine cytology, respectively. Strict follow-up after RNU is more probable recommended for these patients.

## Background

Upper tract urothelial carcinoma (UTUC) is a relatively uncommon condition and accounts for 5–10% of all urothelial malignancies [1]. Nephroureterectomy (NU) with excision of the bladder cuff is the gold standard treatment for non-metastatic UTUC. However, intravesical recurrence (IVR) after NU for UTUC frequently occurs, with an incidence rate of approximately 22–47% [1–4]. Several studies have investigated the risk factors of IVR after NU for UTUC. Reportedly, the risk factors for IVR after NU for UTUC include positive preoperative urine cytology, preoperative diagnostic ureteroscopic biopsy for UTUC, surgery-related factors, such as laparoscopic surgery or endoscopic approach of the bladder cuff excision, lymphovascular invasion (LVI), and concomitant carcinoma in situ (CIS) [4–8].

Recently, laparoscopic NU (LNU) and retroperitoneoscopic NU (RNU) are being performed globally for UTUC. However, there have been discussions about whether LNU and RNU increase the risk of postoperative IVR compared to open NU, and a consensus is yet to be reached [9–13]. On the other hand, few studies have investigated the risk factors of IVR after LNU and RNU, including surgery-related factors.

The purpose of the present study was to investigate the association between IVR after RNU for UTUC and clinicopathological and surgical factors, including preoperative urine cytology, urinary bladder tumor antigen (BTA), urinary nuclear mitotic apparatus protein (NMP22), and pneumoretroperitoneum time.

## Methods

### Patient selection

We retrospectively identified 102 patients treated with RNU for non-metastatic UTUC at Nippon Medical School Hospital between 2012 and 2020. UTUC was diagnosed using computed tomography (CT), magnetic resonance imaging (MRI), and urine cytology. A diagnostic ureteroscopic biopsy was performed when required. All patients underwent preoperative cystoscopy. Of the 102 patients, 29 patients with a history of bladder cancer or concomitant bladder cancer were excluded from our study. Finally, 73 patients were included in the study.

### Clinicopathological data

From the medical records, we collected clinicopathological and surgical information of the patients, including age, sex, laterality and location of the main tumor, presence or absence of hydronephrosis, preoperative urine cytology, preoperative urinary BTA level, preoperative urinary NMP22 level, necessity of diagnostic ureteroscopic biopsy, pneumoretroperitoneum time, total operating time, multifocality of the tumor, tumor size, pathological characteristics, necessity of adjuvant systemic chemotherapy (ASC), and oncological outcomes. Tumors were staged according to the 2002 American Joint Committee of Cancer tumor-node-metastasis classification and were graded according to the 2004 World Health Organization classification [14].

### Surgical procedure

While performing RNU, retroperitoneoscopic procedures were performed in the kidney position, with 8 mmHg CO_2_ gas pressure in all cases. The CO_2_ gas pressure was increased temporally when necessary. The maximum pressure of the CO_2_ gas was 12 mmHg. In the retroperitoneoscopic procedure, we clamped the ureter after ligation of the renal arteries. In all patients, a small iliac incision (Gibson incision) or lower abdominal midline incision was made to retrieve the kidney and ureter and to perform bladder cuff resection with sufficient surgical margin using the extravesical approach. In our institution, we have performed RNU in patients with non-metastatic localized or locally advanced UTUC (cTa-3N0M0). Lymphadenectomy was not performed in this study.

### Adjuvant therapy and follow-up

Adjuvant intravesical therapy is not administered at our institution. Four courses of ASC, such as the gemcitabine/cisplatin regimen or gemcitabine/carboplatin regimen, were administered to select pT2–4 patients. Of these patients, those with an estimated glomerular filtration rate (eGFR) of < 30 ml/min/1.73 m^2^ received ASC with the gemcitabine/carboplatin regimen, and the other patients received ASC with the gemcitabine/cisplatin regimen. After RNU, all patients were generally followed-up using blood tests, urine analysis, urine cytology, cystoscopy, and CT scan every three months for two years, and every six months thereafter. We defined IVR as a pathologically diagnosed bladder cancer after RNU. We also defined progression disease as radiologically diagnosed local or distance recurrence.

### Endpoint of the present study

The primary endpoint of the present study was to investigate the association between IVR after RNU for UTUC and clinicopathological and surgical factors, including preoperative factors of urine cytology, urinary BTA, urinary NMP22, and pneumoretroperitoneum time.

### Statistical analysis

Statistical analyses were performed using JMP® 13 (SAS Institute Inc., Cary, NC, USA). The value of statistical significance was set at *p* < 0.05. The categorical variables were compared using the Fisher exact test and continuous variables using the t-test or the Mann–Whitney U test, depending on the results of the one-sample Kolmogorov–Smirnov test. Survival curves were constructed using the Kaplan–Meier method. To determine the risk factors for IVR in 1 and 3 years after RNU, the Fisher exact test was performed. In the analyses of IVR and progression 1 year after RNU, no IVR and progression cases without 1 year or > 1 year of follow-up were excluded. In the analyses of IVR and progression 3 years after RNU, no IVR and progression cases without 3 years or > 3 years of follow-up were excluded. The cut-off value of the pneumoretroperitoneum time of RNU was 210 min, which was defined as the maximum pneumoretroperitoneum time in the technical certification test of RNU by the Japanese Society of Endourology [15].

## Results

Table 1 demonstrates the characteristics of 73 patients treated with RNU for UTUC. Surgical margins of the bladder cuff were negative in all patients.

**Table 1:** Characteristics of patients treated with RNU for upper urinary tract carcinoma

Preoperative factors		n = 73 (%)
Age (years)	Median (IQR 25–75)	74 (67–79)
Gender	Male/female	56 (76.7)/17 (23.3)
Laterality	Right/left	37 (50.7)/36 (49.3)
Location of main tumor	Ureter/renal pelvis	33 (45.2)/40 (54.8)
Hydronephrosis	Yes/no	24 (32.9)/49 (67.1)
Urine cytology	Positive/negative	32 (43.8)/41 (56.2)
Urinary BTA	Positive/negative	29 (39.7)/44 (60.3)
Urinary NMP22	Positive/negative	36 (49.3)/37 (50.7)
Diagnostic ureteroscopic biopsy	Yes/no	24 (32.9)/49 (67.1)
Intraoperative and postoperative factors		
Pneumoretroperitoneum time (min)	Median (IQR 25–75)	202 (170–268)
	≥ 210/ < 210	32 (43.8)/41 (56.2)
Total operating time (min)	median (IQR 25–75)	352 (302–402)
	≥ 360/ < 360	32 (43.8)/41 (56.2)
Multifocality	multiple/ single	11 (15.1)/62 (84.9)
Tumor size (cm)	≥ 3/ < 3	40 (54.8)/33 (43.8)
Pathological T stage	≤ 1/2/ ≥ 3	30 (41.1)/18 (24.7)/25 (34.2)
Grade	1, 2/3	37 (50.7)/36 (49.3)
LVI	Positive/negative	18 (24.7)/55 (75.3)
INF	a/b, c	21 (28.8)/52 (71.2)
ASC	Yes/ no	23 (31.5)/50 (68.5)

*IQR* interquartile range, *BTA* bladder tumor antigen, *NMP22* nuclear mitotic apparatus protein, *LVI* lymphovascular invasion, *INF* infiltrative growth, *ASC* adjuvant systemic chemotherapy

**Fig. 1:**
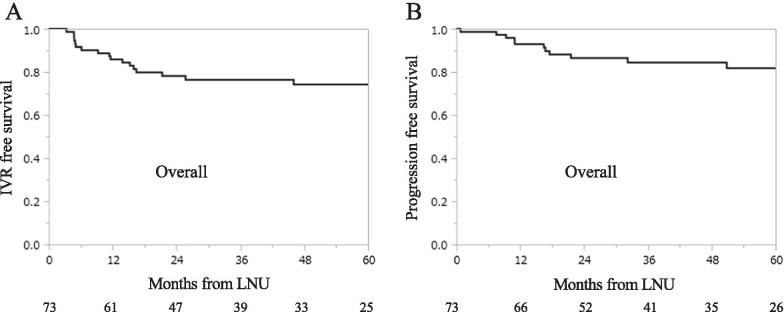
IVR-free survival and PFS in 73 patients.** A** Kaplan–Meier curves of IVR-free survival in 73 patients.** B** Kaplan–Meier curves of PFS in 73 patients

During the median follow-up of 39.1 months after RNU, 18 (24.7%) patients had IVR. The 1-year and 3-year IVR-free survival rates were 85.9% and 76.5%, respectively (Fig. 1A). The histological type of bladder cancer in 18 patients was urothelial carcinoma. Table 2 demonstrates the multifocality and location of IVR tumors. In 50% of these bladder cancers, the grade was lower than that of the initial UTUC diagnosis. In the other 50% of bladder cancer cases, the grade was the same grade as that of the initial UTUC. None of the bladder cancers had a higher grade than the initial UTUC diagnosis.

**Table 2:** Multifocality and location of intravesical recurrence tumors

	n = 18 (%)
Multifocality	
Single	14 (77.8)
Multiple	4 (22.2)
Tumor location	
Scar site	11 (61.1)
Bladder neck	5 (27.8)
Lateral wall	4 (22.2)
Dome	1 (5.5)
Posterior wall	1 (5.5)

The Fisher exact test revealed that prolonged pneumoretroperitoneum time of ≥ 210 min was a risk factor for IVR in 1 year after RNU (*p* = 0.0358) (Table 3) and positive urine cytology was a risk factor for IVR in 3 years after RNU (*p* = 0.0352) (Table 4).

**Table 3:** Risk factor analysis of IVR in 1 year after RNU

Variables		No IVR n = 60 (%)	IVR n = 10 (%)	*p*-value
Age (years)	≥ 70	42 (70.0)	5 (50.0)	0.2790
Gender	Male	45 (75.0)	8 (80.0)	1.0000
Laterality	Right	32 (53.3)	4 (40.0)	0.5079
Location of main tumor	Ureter	25 (41.7)	6 (60.0)	0.3203
Hydronephrosis	Yes	19 (31.7)	3 (30.0)	1.0000
Urine cytology	Positive	24 (40.0)	7 (70.0)	0.0956
Urinary BTA	Positive	22 (36.7)	6 (60.0)	0.1833
Urinary NMP22	Positive	28 (46.7)	5 (50.0)	1.0000
Diagnostic ureteroscopic biopsy	Yes	18 (30.0)	5 (50.0)	0.2790
Multifocality	Multiple	6 (10.0)	3 (30.0)	0.1120
Tumor size (cm)	≥ 3	29 (48.3)	3 (30.0)	0.3263
Pathological T stage	≥ 3	19 (31.7)	5 (50.0)	0.2945
Grade	3	29 (48.3)	5 (50.0)	1.0000
LVI	Positive	15 (25.0)	2 (20.0)	1.0000
INF	b, c	41 (68.3)	9 (90.0)	0.2618
Pneumoretroperitoneum time (min)	≥ 210	24 (40.0)	8 (80.0)	0.0358*
Total operating time (min)	≥ 360	25 (41.7)	6 (60.0)	0.3203
ASC	Yes	19 (31.7)	4 (40.0)	0.7192

*IVR* intravesical recurrence, *BTA* bladder tumor antigen, *NMP22* nuclear mitotic apparatus protein, *LVI* lymphovascular invasion, *INF* infiltrative growth, *ASC* adjuvant systemic chemotherapy

*p < 0.05

**Table 4:** Risk factor analysis of IVR in 3 years after RNU

Variables		No IVR n = 38 (%)	IVR n = 16 (%)	*p*-value
Age (years)	≥ 70	26 (68.4)	7 (43.8)	0.1280
Gender	Male	29 (76.3)	12 (75.0)	1.0000
Laterality	Right	18 (47.3)	7 (43.8)	1.0000
Location of main tumor	Ureter	18 (47.3)	8 (50.0)	1.0000
Hydronephrosis	Yes	11 (28.9)	6 (37.5)	0.5402
Urine cytology	Positive	15 (39.5)	12 (75.0)	0.0352*
Urinary BTA	Positive	15 (39.5)	9 (56.3)	0.3695
Urinary NMP22	Positive	17 (44.7)	8 (50.0)	0.7718
Diagnostic ureteroscopic biopsy	Yes	12 (31.6)	8 (50.0)	0.2301
Multifocality	Multiple	3 (7.9)	3 (18.8)	0.3461
Tumor size (cm)	≥ 3	16 (42.1)	7 (43.8)	1.0000
Pathological T stage	≥ 3	11 (28.9)	6 (37.5)	0.5402
Grade	3	16 (42.1)	9 (56.3)	0.3836
LVI	Positive	7 (18.4)	5 (31.3)	0.3090
INF	b, c	27 (71.1)	13 (81.3)	0.5155
Pneumoretroperitoneum time (min)	≥ 210	16 (42.1)	11 (68.8)	0.1350
Total operating time (min)	≥ 360	16 (42.1)	10 (62.5)	0.2358
ASC	Yes	13 (34.2)	5 (31.3)	1.0000

*IVR* intravesical recurrence, *BTA* bladder tumor antigen, *NMP22* nuclear mitotic apparatus protein, *LVI* lymphovascular invasion, *INF* infiltrative growth; ASC, adjuvant systemic chemotherapy

*p < 0.05

Table 5 demonstrates two group analyses based on the pneumoretroperitoneum time of 210 min to investigate the presence of bias. No significant difference between these two groups was noted.

**Table 5:** The characteristics of patients according to pneumoretroperitoneum time

Variables		PT < 210 min n = 41 (%)	PT ≥ 210 min n = 32 (%)	*p*-value
Age (years)	Median (IQR 25–75)	75 (67–80)	74 (67–78)	0.4168
Gender	Male	32 (78.0)	24 (75.0)	0.7865
Laterality	Right	18 (43.9)	19 (59.4)	0.2405
Location of main tumor	Ureter	19 (46.3)	14 (43.8)	1.0000
Hydronephrosis	Yes	15 (36.6)	9 (28.1)	0.4655
Urine cytology	Positive	15 (36.6)	17 (53.1)	0.2345
Urinary BTA	Positive	12 (29.3)	17 (53.1)	0.1496
Urinary NMP22	Positive	20 (48.8)	16 (50.0)	1.0000
Diagnostic ureteroscopic biopsy	Yes	15 (36.6)	9 (28.1)	0.4655
Multifocality	Multiple	6 (14.6)	5 (15.6)	1.0000
Tumor size (cm)	≥ 3	19 (46.3)	14 (43.8)	1.0000
Pathological T stage	≥ 3	15 (36.6)	10 (31.3)	0.8041
Grade	3	22 (53.7)	14 (43.8)	0.4818
LVI	Positive	10 (24.3)	8 (25.0)	1.0000
INF	b, c	30 (73.2)	22 (68.8)	0.7958
ASC	Yes	13 (31.7)	10 (31.3)	1.0000

*PT* pneumoretroperitoneum time, *IQR* interquartile range, *BTA* bladder tumor antigen, *NMP22* nuclear mitotic apparatus protein, *LVI* lymphovascular invasion, *INF* infiltrative growth, *ASC* adjuvant systemic chemotherapy

Of the 73 cases, 15 (20.5%) were positive for urine cytology, NMP22, and BTA, and 18 (24.7%) were positive for two of these three.

During the median follow-up of 41.9 months after LNU, 12 (16.4%) patients had a metastatic recurrence. The 1-year and 3-year progression-free survival (PFS) rates were 92.9% and 84.5%, respectively (Fig. 1B). The Fisher exact test revealed that pathological T ≥ 3 was a risk factor for progression in 1 year after RNU (p = 0.0439) (Table 6), and pathological T ≥ 3 (*p* = 0.0007), Grade 3 (*p* = 0.0145), LNI (*p* = 0.0073), and ASC (*p* = 0.0088) were the risk factors for progression in 3 years after RNU (Table 7).

**Table 6:** Risk factor analysis of progression in 1 year after RNU

Variables		No progression n = 65 (%)	Progression n = 5 (%)	*p*-value
Age (years)	≥ 70	42 (64.6)	5 (100.0)	0.1639
Gender	Male	48 (73.8)	5 (100.0)	0.3255
Laterality	Right	33 (50.8)	3 (60.0)	1.0000
Location of main tumor	Ureter	28 (43.1)	3 (60.0)	0.6489
Hydronephrosis	Yes	20 (30.8)	2 (40.0)	0.6463
Urine cytology	Positive	31 (47.7)	0 (0.0)	0.0616
Urinary BTA	Positive	27 (41.5)	1 (20.0)	0.6415
Urinary NMP22	Positive	32 (49.2)	1 (20.0)	0.3608
Diagnostic ureteroscopic biopsy	Yes	21 (32.3)	2 (40.0)	1.0000
Multifocality	Multiple	9 (13.8)	0 (0.0)	1.0000
Tumor size (cm)	≥ 3	28 (43.1)	4 (80.0)	0.1710
Pathological T stage	≥ 3	20 (30.8)	4 (80.0)	0.0439*
Grade	3	30 (46.2)	4 (80.0)	0.1921
LVI	Positive	15 (23.1)	2 (40.0)	0.5887
INF	b, c	45 (69.2)	5 (100.0)	0.3117
Pneumoretroperitoneum time (min)	≥ 210	30 (46.2)	2 (40.0)	1.0000
Total operating time (min)	≥ 360	29 (44.6)	2 (40.0)	1.0000
ASC	Yes	20 (30.8)	3 (60.0)	0.3221

*BTA* bladder tumor antigen, *NMP22* nuclear mitotic apparatus protein, *LVI* lymphovascular invasion, *INF* infiltrative growth, *ASC* adjuvant systemic chemotherapy

*p < 0.05

**Table 7:** Risk factor analysis of progression in 3 years after RNU

Variables		No progression n = 40 (%)	Progression n = 10 (%)	*p*-value
Age (years)	≥ 70	24 (60.0)	7 (70.0)	0.7222
Gender	Male	29 (72.5)	8 (80.0)	1.0000
Laterality	Right	17 (42.5)	6 (60.0)	0.4804
Location of main tumor	Ureter	19 (47.5)	6 (60.0)	0.7252
Hydronephrosis	Yes	12 (30.0)	3 (30.0)	1.0000
Urine cytology	Positive	19 (47.5)	5 (50.0)	1.0000
Urinary BTA	Positive	18 (45.0)	3 (30.0)	0.4880
Urinary NMP22	Positive	18 (45.0)	5 (50.0)	1.0000
Diagnostic ureteroscopic biopsy	Yes	12 (30.0)	3 (30.0)	1.0000
Multifocality	Multiple	4 (10.0)	0 (0.0)	0.5710
Tumor size (cm)	≥ 3	14 (35.0)	8 (80.0)	0.0836
Pathological T stage	≥ 3	8 (20.0)	8 (80.0)	0.0007*
Grade	3	22 (55.0)	9 (90.0)	0.0145*
LVI	Positive	6 (15.0)	6 (60.0)	0.0073*
INF	b, c	28 (70.0)	10 (100.0)	0.0920
Pneumoretroperitoneum time (min)	≥ 210	20 (50.0)	5 (50.0)	1.0000
Total operating time (min)	≥ 360	19 (47.5)	5 (50.0)	1.0000
ASC	Yes	12 (30.0)	8 (80.0)	0.0088*

*BTA* bladder tumor antigen, *NMP22* nuclear mitotic apparatus protein, *LVI* lymphovascular invasion, *INF* infiltrative growth, *ASC* adjuvant systemic chemotherapy

*p < 0.05

## Discussion

LNU is the mainstream surgery for UTUC and RNU is not popularly performed [8, 16, 17]. Therefore, most studies are focused on LNU. Here, we focused on RNU. This is the first report investigating the risk factors, including the pneumoretroperitoneum time, for IVR after RNU.

In this study, prolonged pneumoretroperitoneum time of ≥ 210 min was a risk factor for IVR in 1 year after RNU, with 8 mmHg CO_2_ gas pressure (Table 3). In a previous study, Shigeta et al. reported that prolonged pneumoperitoneum time of LNU for UTUC was an independent risk factor for IVR [8]. They performed LNU (62.8%) and RNU (37.2%) for their cohort. The results of the present and the previous studies suggested that CO_2_ gas pressure time impact on IVR. Shigeta et al. analyzed a cohort similar to this study that excluded patients with a history of bladder cancer or concomitant bladder cancer; the IVR rate was 47.3% during the median follow-up of 31.1 months postoperatively. They performed LNU or RNU with 10 mmHg CO_2_ gas pressure and the median pneumoperitoneum or pneumoretroperitoneum time was 150 min; meanwhile, the median pneumoretroperitoneum time of the present study was 202 min, which was significantly longer than that of their study. However, in the present study, the IVR rate after RNU was 24.7% during the median follow-up of 39.1 months postoperatively, which was significantly lower than that of the study by Shigeta et al. When the two studies were compared, the differences were observed in CO_2_ gas pressure and surgical procedure. It was suggested that a low CO_2_ gas pressure of 8 mmHg and/or RNU in the present study might have influenced the low IVR rates. Further studies with large cohorts comparing different CO_2_ gas pressures are needed to investigate the impact of CO_2_ gas pressure on IVR postoperatively. Moreover, we only analyzed RNU in this study; therefore, it remains unclear whether the results of this study apply to LNU, because the pressure on the ureter during surgery might be different between RNU and LNU. Further studies comparing RNU and LNU are required.

This study was a retrospective study without a pilot study. The study began in February 2021, and the results were disclosed to all urologists at our institution in April 2021. Therefore, there was no bias of knowledge in the study results. We also analyzed the factors related to prolonged pneumoretroperitoneum time. However, any factors related to prolonged pneumoretroperitoneum time were not present (Table 5).

Recent molecular genetic studies have suggested that intraluminal seeding is one of the main mechanisms of IVR after NU [18–20]. It was also reported that continuous intravesical irrigation with distilled water or physiological saline solution during LNU decreased the rate of IVR incidence [16]. They concluded that continuous intravesical irrigation might eliminate cancer cells floating in the bladder during surgery before they become engrafted on the mucous membrane of the bladder. This result suggests that IVR after NU occurs due to intraluminal seeding. Recent studies demonstrated that prolonged CO_2_ gas pressure time and diagnostic ureteroscopic biopsy are independent factors of IVR after NU [5, 8]. Based on these results of past studies, long-term CO_2_ gas pressure to the tumor and direct destruction of the tumor by diagnostic ureteroscopic biopsy might contribute to intraluminal seeding. In the present study, the grade of bladder cancer with IVR was not higher than that of initial UTUC. It has also been suggested that IVR tumors are caused by intraluminal seeding from UTUC.

The BTA test detects the human complement factor H-related protein secreted in the urine. While the NMP22 test detects the protein level of the nuclear mitotic apparatus. Positive urinary BTA and NMP22 have been reported as predictors of the presence of bladder cancer and UTUC, along with positive urine cytology [21–24]. In the present study, the risk factor for IVR 3 years after RNU was not positive urinary BTA or urinary NMP22, but positive urine cytology (Table 4). Additionally, only 20.5% of patients had all three positive urine cytology, urinary BTA, and urinary NMP22. There is not much overlap between urine cytology, urinary BTA, and urinary NMP22. Urinary BTA and NMP22 are considered unsuitable for predicting IVR after RNU because the values of urinary BTA and NMP22 generally have a positive correlation with tumor volume; however, urinary BTA and NMP22 do not directly detect cancer cells.

Physical injury to the bladder is associated with increased adherence of tumor cells to the urothelium [25]. In NU, the bladder cuff and ureteral orifice are resected, while a urethral catheter is maintained in the bladder during and several days after NU. Physical injury to the urothelium, such as bladder cuff resection and stimulation of the bladder mucosa with a ureteral catheter, might support the growth of tumor cells in the urothelium [26]. In the present study, 11 (61.1%) and 5 (27.8%) patients had IVR tumors located at scar site and bladder neck, which could have been stimulated by the urethral catheter (Table 2). Hence, this result is consistent with the that of the previous study [26].

Recently, two prospective randomized trials have demonstrated that a single early intravesical chemotherapy cycle using mitomycin C or pirarubicin after NU decreased the risk of IVR [27, 28]. However, the type of patients that will benefit from this treatment remains unclear. In this study, prolonged pneumoretroperitoneum time of ≥ 210 min was a risk factor for IVR in 1 year after RNU and positive urine cytology was a risk factor for IVR in 3 years after RNU. From our results, we strongly recommend that patients with pneumoperitoneum time of ≥ 210 min and/or with positive urine cytology should receive a single early intravesical chemotherapy after RNU with 8 mmHg CO_2_ gas pressure.

In this study, pathological findings of UTUC were the risk factors for progression after RNU, not pneumoretroperitoneum time (Tables 6, 7). Therefore, when the pneumoretroperitoneum time of RNU is prolonged, an attending physician can perform a follow-up imaging after RNU at normal intervals.

The present study has several limitations. UTUC is a relatively uncommon condition. We excluded patients with a history of bladder cancer or concomitant bladder cancer, because the purpose of the present study was to investigate the risk factors for IVR after RUN for UTUC. In addition, this study was conducted in a single institution; therefore, the cohort in this study was small. Since the study was a retrospective analysis, there might be a selection bias for the surgeons. In this study, 13 surgeons performed the RUN procedure. However, three experienced surgeons who had performed more than 100 laparoscopic surgeries performed or supervised all of the RUN procedures. In addition, the rate of IVR incidence in our study was lower than that reported in previous studies. Based on these facts, we believe that the participation of inexperienced surgeons in RUN had little impact on the IVR rate in the present study. To reduce these limitations, prospective studies with larger cohorts from several institutions are required. Currently, lymphadenectomy is recommended for pathological T ≥ 2 UTUC. However, lymphadenectomy was not performed in this study. The reasons are that there are several discrepancies between the clinical T stage and the pathological T stage, and there are technical issues with retroperitoneoscopic lymphadenectomy. The lack of lymphadenectomy in this study might impact on PFS. In our institution, we performed open NU and lymphadenectomy only in cases suspected of visible lymph node metastasis on CT. There is an urgent need to improve the accuracy of diagnostic imaging for staging and lymphadenectomy for clinical T ≥ 2 UTUC. Finally, the risks of IVR logically related to the time from infusing CO_2_ gas pressure to the clipping of the ureter. However, we were only able to collect the data on clipping time of the ureter for some patients using their operation and intraoperative nursing records. Therefore, it was difficult to analyze association between IVR after RNU and the time from infusing CO_2_ gas pressure to the clipping of the ureter. Further studies analyzing the association between IVR after RNU and the time from infusing CO_2_ gas pressure to the clipping of the ureter are required.

## Conclusions

In UTUC, the occurrence of IVR in 1 year after RNU is highly probable when the pneumoretroperitoneum time is prolonged (≥ 210 min) and the occurrence of IVR in 3 years after RNU is highly probable in patients with positive urine cytology. Strict follow-up after RNU is more probable recommended for these patients.

## Data Availability

The datasets used and/or analyzed during the current study are available from the corresponding author upon reasonable request.

## Abbreviations

ASC: Adjuvant systemic chemotherapy
BTA: Bladder tumor antigen
CIS: Carcinoma in situ
CT: Computed tomography
eGFR: Estimated glomerular filtration rate
INF: Infiltrative growth
IVR: Intravesical recurrence
LNU: Laparoscopic nephroureterectomy
LVI: Lymphovascular invasion
NMP22: Nuclear mitotic apparatus protein 22
NU: Nephroureterectomy
PFS: Progression-free survival
RNU: Retroperitoneoscopic nephroureterectomy
UTUC: Upper tract urothelial carcinoma
